# Effect of Deep-Level Defects on the Performance of CdZnTe Photon Counting Detectors

**DOI:** 10.3390/s20072032

**Published:** 2020-04-04

**Authors:** Yingrui Li, Gangqiang Zha, Dengke Wei, Fan Yang, Jiangpeng Dong, Shouzhi Xi, Lingyan Xu, Wanqi Jie

**Affiliations:** 1 State Key Laboratory of Solidification Processing, and MIIT Key Laboratory of Radiation Detection Materials and Devices, Northwestern Polytechnical University, Xi’an 710072, China; li_yrui@foxmail.com (Y.L.); weidk@nwpu.edu.cn (D.W.); yang2012100392@163.com (F.Y.); dong_jp@mail.nwpu.edu.cn (J.D.); xiaola507@163.com (S.X.); xulingyan@nwpu.edu.cn (L.X.); jwq@nwpu.edu.cn (W.J.); 2 Ametek Co., Ltd., Shaanxi Xixian New Area Qinhan New City Tian Gong 1 road 8-1, Xi’an 712000, China

**Keywords:** CdZnTe, deep-level defects, photon counting performance, polarization effect, defect reaction

## Abstract

The effect of deep-level defects is a key issue for the applications of CdZnTe high-flux photon counting devices of X-ray irradiations. However, the major trap energy levels and their quantitive relationship with the device’s performance are not yet clearly understood. In this study, a 16-pixel CdZnTe X-ray photon counting detector with a non-uniform counting performance is investigated. The deep-level defect characteristics of each pixel region are analyzed by the current–voltage curves (I–V), infrared (IR) optical microscope photography, photoluminescence (PL) and thermally stimulated current (TSC) measurements, which indicate that the difference in counting performance is caused by the non-uniformly distributed deep-level defects in the CdZnTe crystals. Based on these results, we conclude that the CdZnTe detectors with a good photon counting performance should have a larger ${\mathrm{Te}}_{\mathrm{cd}}^{2+}$ and Cd vacancy-related defect concentration and a lower A-center and Te_i_ concentration. We consider the deep hole trap Te_i_, with the activation energy of 0.638–0.642 eV, to be the key deep-level trap affecting the photon counting performance. In addition, a theoretical model of the native defect reaction is proposed to understand the underlying relationships of resistivity, deep-level defect characteristics and photon counting performance.

## 1. Introduction

There is growing interest in the potential of direct converting semiconductor detectors for the detection of X-rays and γ-rays [1,2,3]. Owing to its wide band-gap, high resistivity, high density and atomic number and excellent carrier transport properties, semi-insulating cadmium zinc telluride (CdZnTe) is widely considered as a very suitable material for energy-resolved X-ray photon counting devices [4]. The high-flux, multi-energy binning X-ray imaging CdZnTe detector technology, based on the pulse mode, has become a research hotspot [5,6,7]. These large-volume, good-uniformity CdZnTe photon counting detectors, which can detect a photon flux in the order of a hundred million mm^−2^s^−1^, are necessary for many applications such as medical and industrial imaging [8,9]. Although such high-flux CdZnTe detectors have been reported [10], it is still a challenge to achieve large-scale commercial applications, primarily due to the material defects which can lead to the polarization effect [11,12,13,14,15,16]. Under high X-ray flux conditions, excessive positive space charges formed by trapped holes build up inside the detector, which have an opposite effect on the externally applied voltage and ultimately result in a non-uniform electric field and catastrophic device failure. During the growth process of CdZnTe crystals, the material defects such as Cd vacancies, Te antisites, Te interstitials, Cd vacancy–Te antisite pairs and Cd vacancy complexes (A-centers) will inevitably be introduced, and they are very important in achieving a high resistivity. The polarization effect is associated with the presence of these material defects, especially deep-level defects [15,17], which can strongly affect the carrier transport properties. Therefore, it makes sense to determine the correlation between the crystal defects and the performance of CdZnTe photon counting detectors, which will be helpful in guiding the crystal growth technique and in developing the utilization of large-area low-cost photon counting devices. Several related simulation and experimental studies have been reported on the coupling relationship among crystal defects, the photon counting performance and the polarization effect of CdZnTe detectors [18,19,20]. However, the major trap energy levels and their origins affecting photon counting performance are still controversial.

In this paper, 16-pixel CdZnTe photon counting detectors with a non-uniform counting performance are adopted for the experimental study. The characteristics of the deep-level defects are obtained by the current–voltage curves (I–V), infrared optical microscope photography, photoluminescence (PL) and thermally stimulated current (TSC) measurements. A theoretical model of the native defect reaction is proposed to analyze the underlying relationship among the resistivity, deep-level defects and photon counting performance. Eventually, the key deep-level trap that affects the photon counting performance will be assigned, which may provide ideas for the CdZnTe crystal growth technique for future photon counting applications.

## 2. Experimental Section

**Figure 1:**
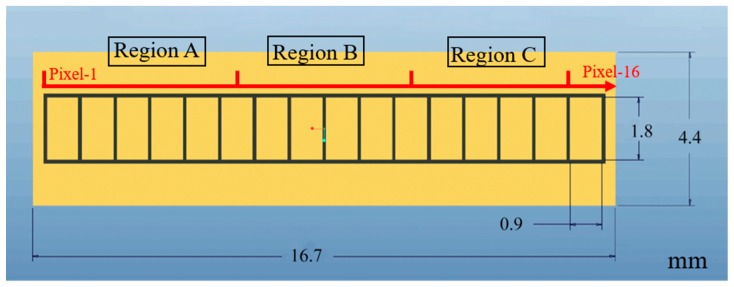
Schematic diagram of the detailed anode structure containing the location information of pixel 1 to 16 and region A to C.

To explore the relationship between the deep-level defects and performance of CdZnTe photon counting detectors, and to exclude the influence of other factors such as the processing technique, indium-doped Cd_0.9_Zn_0.1_Te wafers with non-uniformly distributed defects, grown by the modified vertical Bridgman (MVB) method from Imdetek Ltd., were selected for preparing the 16-pixel CdZnTe photon counting detectors. The dimensions of the wafers were 16.6 × 4.4 × 2 mm. After polishing and etching, Au electrodes were fabricated on both the anode and cathode surfaces by the vacuum evaporation deposition method. The detector cathode is a planar electrode. The detailed anode structure is shown in Figure 1. The anode of the CdZnTe detector consists of pixels with an area of 0.9 × 1.8 mm. The count rate data were collected via a 16-channel application-specific integrated circuit (ASIC) readout system, which is manufactured by Imdetek Ltd. This ASIC readout system was described in detail in [17]. The X-ray source used in the measurement was the Spellman XRB011, which can achieve a maximum tube current of 0.6 mA at 80 kV. An Agilent 6517B electrometer was employed for the I–V analysis to determine the local resistivity of each pixel. The point defect levels were determined using PL technology which can achieve a 0.5 nm spectral resolution. In the PL measurements, an argon ion laser was used as the excitation source, with a power of 20 mW and a wavelength of 488 nm. Immediately after it was etched by the Everson solution, the dislocation etch pit density (EPD) on the CdZnTe {111} Te face of each pixel region was observed using infrared (IR) transmission microscopy. The TSC measurement was carried out to obtain the concentration and distribution information of the deep-level traps in the CdZnTe crystals. The sample was cooled down to 80 K in a closed-cycle cryostat in the darkness, and then free carriers excited by the 650 nm light of the intensity of ~2.5μW/cm^2^ from a halogen lamp would be captured by the defect traps. As the temperature rose to 320 K at a rate of 0.2 K/s, the current signal caused by the thermal excitation of the trapped electrons and holes was recorded under a 10 V applied bias by a Keithley 6514 electrometer.

## 3. Results and Discussion

### 3.1. X-ray Photon Counting Performance

**Figure 2:**
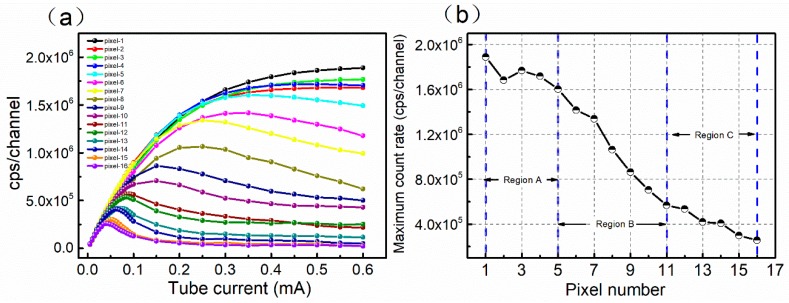
(**a**) Count rate performance of the 16-pixel CdZnTe photon counting detector; (**b**) trend of the maximum count rate of the 16 pixels. The regions A, B and C represent the high counting performance region, the medium counting performance region and the poor counting performance region.

Figure 2a shows the photon counting performance of the 16-pixel CdZnTe detector. The tube voltage was fixed at 80 kV and the tube current was changed from 0 to 0.6 mA. The photon counting response of the 16-pixel detectors exhibits a regular non-uniformity, i.e., the counting performance decreases in order (pixel-1 to pixel-16). As the tube current increases, the counting rate curves of some pixels, such as pixel-1, first increase linearly, then deviate from linearity and reach a saturation value, mainly limited by the electronic dead time and pulse pile-up effect. For other pixels, as the current tube gradually increases, the count rate first increases, then the count rate curves show a downward trend and the count rate almost drops to zero in extreme conditions such as pixel-16. The detector polarization should be responsible for the reduction of the count rate [15]. This indicates that a large number of free holes excited by the X-ray photons are captured by defect traps due to the poor hole transport properties. The trapped holes can build up in the crystal to form positive space charges and ultimately deteriorate the photon counting performance of the detector. It should be noticed that the maximum count rate decreases from pixel-1 to pixel-16 in sequence, as shown in Figure 2b, which indicates that there are differences in the defect characteristics at different pixel regions of the CdZnTe detector.

### 3.2. Deep-Level Defect Characteristics

**Figure 3:**
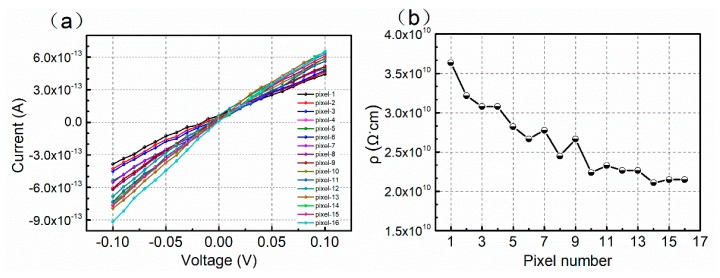
(**a**) Current–voltage (I–V) curves for the 16 pixel regions of the CdZnTe photon counting detector; (**b**) trend of resistivity of the 16 pixels.

In order to determine the potential causes of the discrepancies in the count rate, the bulk resistivity for all the 16 pixel regions was evaluated by an I–V measurement at room temperature. The bulk resistivity, *ρ*, is defined by

(1) $$\begin{array}{c} \rho =\frac{U}{I}\frac{S}{d} \end{array}$$

where *S* is the electrode area and *d* is the thickness of the detector. All the I–V curves of the 16 pixels are very linear in the voltage range of −0.1 V to 0.1 V, as shown in Figure 3a. All pixel regions show a high resistivity (>10^10^ Ω·cm) which is beneficial for the applications of CdZnTe photon counting detectors. The high resistivity means that the detectors can withstand a higher applied bias, thus increasing the carrier collection efficiency (CCE). Moreover, the low leakage current can reduce the burden on the ASIC readout electronics [10]. The resistivity differences of the different pixels are shown in Figure 3b. Compared with Figure 2, the pixels with a higher resistivity show a better photon counting performance. The resistivity is closely related to the defect characteristics in CdZnTe crystals. Existing research has focused on the high resistivity mechanism [21,22]. The resistivity of CdZnTe semiconductors is closely related to the crystal defect characteristics. Indium doping, introducing shallow levels only, can effectively compensate for the acceptors, resulting in high resistivity. Fan et al. proposed that the difference in indium doping may lead to changes in resistivity by changing the difference between the conduction band edge and Fermi level [23]. Deep-level defects are also essential to achieve high-resistance CdZnTe crystals, which can effectively reduce the free carrier concentration. We can, therefore, conclude that the different defect characteristics should be responsible for the differences in resistivity and counting performance.

**Figure 4:**
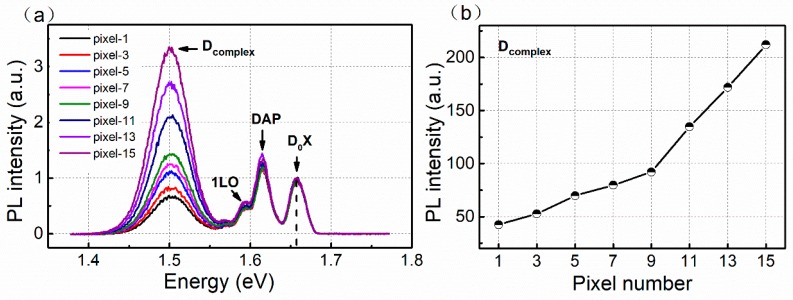
(**a**) Photoluminescence (PL) spectra from the typical pixels with different photon counting performance; (**b**) The values of the D_complex_ peak intensity of the typical pixels.

To further understand the influence of defects on the photon counting performance, the studied 16-pixel CdZnTe detector was mechanically and chemically polished, then etched with a 2% Br/methanol solution prior, again, to PL measurements. Figure 4a shows the PL spectra of the typical pixel regions at 10 K. For comparison, the horizontal axis has been normalized with the bound exciton peaks (D^0^X) to show the energy shift relative to the bandgap. Three main energy levels in the PL spectra are clearly visible for all 16 pixel regions, namely the bound exciton peaks (D^0^X) located at around 1.657 eV, the donor–acceptor pair (DAP) recombination and its phonon replicas (1LO) located at around 1.594 eV and a defect band called the D_complex_, which is related to the Cd vacancy in the complex and dislocations, with peaks centered at around 1.501 eV. From the analysis on the PL data, we can conclude the following: First, the (D^0^X) peaks, which are thought to be dependent on the Zn concentration according to [24], are essentially the same for all 16 pixels. This means that the Zn inhomogeneous distribution cannot be the reason for the different photon counting performances. Secondly, the position and intensity of the DAP peaks are also almost the same. This means that there are no strong fluctuations in the related ionized donors and acceptors in the bandgap. Thirdly, the peak intensities of the D_complex_ of the different pixel regions show a large difference, which indicates the non-uniformly distribution of the related defects. As shown in Figure 4b, the pixels with a good counting performance have a lower D_complex_ intensity.

**Figure 5:**
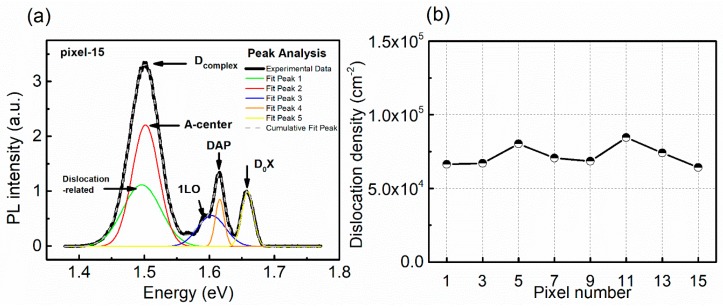
(**a**) Multi-Gaussian fitting for the typical PL spectra using pixel-15 as an example; (**b**) statistical results of the dislocation density in the typical pixel regions.

**Figure 6:**
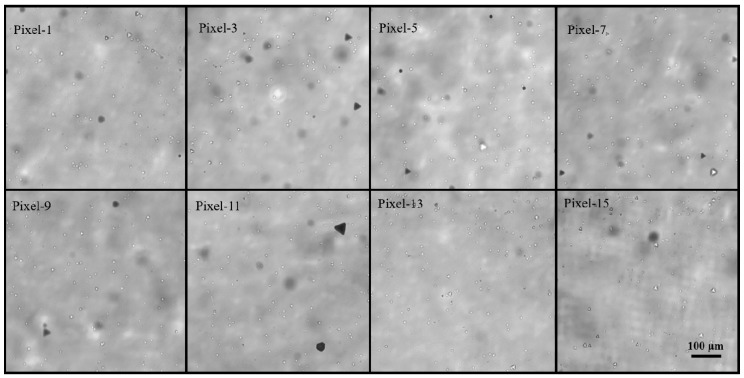
IR transmission microscopy images of the dislocation etch pits at the typical pixel regions of 16-pixel photon counting detectors. Note that the observation face is the {111} Te face and the dislocation etchant is the Everson solution.

As mentioned in many related studies [25,26], the D_complex_ contains the information of the dislocations and so-called A-center attributed to ${\left[{\mathrm{In}}_{\mathrm{Cd}}^{+}V_{\mathrm{Cd}}^{2-}\right]}^{-}$ in the CdZnTe crystals, which can be distinguished by fitting the PL spectra with Gaussian shapes, as shown in Figure 5a. To exclude the possibility that the differences in the D_complex_ peak intensity are caused by dislocations, IR transmission microscopy was carried out to obtain the dislocation etch pit density (EPD) of the typical pixel regions, as shown in Figure 6. The uniformly distributed dislocation etch pits with a density of (6.4–8.5) × 10^5^ cm^−2^ were observed at all pixel regions. The statistical results in Figure 5b show that the EPD of each pixel region is in the same order of magnitude with little difference, which indicates that it is the A-center, rather than the dislocations, that causes the different D_complex_ peak intensities in the PL spectra. We can conclude that the photon counting performance is mainly determined by the A-centers, and the pixels with a good counting performance should have a lower A-center concentration.

The following defect reactions may occur during the growth in Te-rich conditions and the annealing process of the indium-doped CdZnTe crystals:

(2) $$\begin{array}{c} \mathrm{In}+V_{\mathrm{cd}}^{2-}\to {\mathrm{In}}_{\mathrm{cd}}^{+}+3e^{-} \end{array}$$

(3) $$\begin{array}{c} {\mathrm{In}}_{\mathrm{cd}}^{+}+V_{\mathrm{cd}}^{2-}\to {\left[{\mathrm{In}}_{\mathrm{cd}}^{+}V_{\mathrm{cd}}^{2-}\right]}^{-} \end{array}$$

(4) $$\begin{array}{c} V_{\mathrm{cd}}^{2-}+{\mathrm{Te}}_{i}\to {\mathrm{Te}}_{\mathrm{cd}}^{2+}+4e^{-} \end{array}$$

Indium, as a kind of shallow donor impurity, is intentionally introduced to compensate for a Cd vacancy, which is considered to be an intrinsic acceptor. Indium can occupy a Cd vacancy and form ${\mathrm{In}}_{\mathrm{cd}}^{+}$, which is considered as a shallow donor state and can increase the free electron concentration in the conduction band. Compared with the resistivity measurement results, the pixels with a good counting performance have a higher resistivity, which may be dependent on their lower ${\mathrm{In}}_{\mathrm{cd}}^{+}$ concentration. Subsequently, ${\mathrm{In}}_{\mathrm{cd}}^{+}$ can continue to react with $V_{\mathrm{cd}}^{2-}$ to form ${\left[{\mathrm{In}}_{\mathrm{cd}}^{+}V_{\mathrm{cd}}^{2-}\right]}^{-}$, the so-called A-center, as in Formula (2). Therefore, the concentration of the A-center and ${\mathrm{In}}_{\mathrm{cd}}^{+}$ is positively correlated. The PL test results are in good agreement with the fact that the pixel regions with a higher resistivity are always synchronized with the lower A-center concentrations. The lower ${\mathrm{In}}_{\mathrm{cd}}^{+}$ and A-center concentrations mean that more $V_{\mathrm{cd}}^{2-}$ can remain to react with Te_i_ to form ${\mathrm{Te}}_{\mathrm{cd}}^{2+}$, resulting in a decrease in the Te_i_ concentration and an increase in the ${\mathrm{Te}}_{\mathrm{cd}}^{2+}$ concentration, as in Formula (3). It is worth noting that ${\mathrm{Te}}_{\mathrm{cd}}^{2+}$ is generally considered as a deep electron trap which can reduce the concentration of free carriers and strongly deteriorate the electron transport properties [22], while Te_i_ is a deep hole trap [27] that will affect the hole’s transport properties, which is closely related to the counting performance. Based on the above analysis, we imply that CdZnTe detectors with a good counting performance have lower ${\mathrm{In}}_{\mathrm{cd}}^{+}$, ${\left[{\mathrm{In}}_{\mathrm{cd}}^{+}V_{\mathrm{cd}}^{2-}\right]}^{-}$ and Te_i_ concentrations and a higher ${\mathrm{Te}}_{\mathrm{cd}}^{2+}$ concentration. The above results agree well with [11], that the high-flux CdZnTe detectors have a higher hole mobility-lifetime product and lower electron mobility-lifetime product.

**Figure 7:**
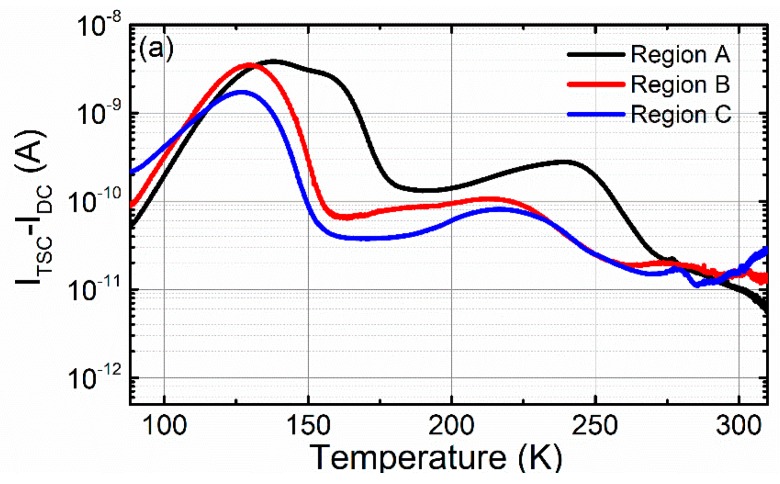

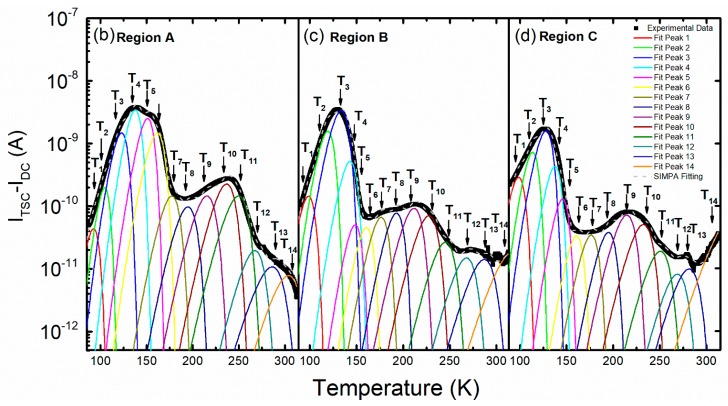
Thermally stimulated current (TSC) spectra with the simultaneous multiple peak analysis (SIMPA) fitting results of the three typical regions in the experimental CdZnTe sample. (**a**) The overview of TSC spectra; (**b**–**d**) the SIMPA fitting results of region A, region B and region C, respectively.

In order to clarify the influence of deep-level defects on the photon counting performance, the TSC measurement was carried out to obtain the trap’s information in the 16-pixel CdZnTe detector. For comparison, the sample was divided into three regions, namely region A, region B and region C, as marked in Figure 1, which respectively correspond to the high counting performance region, the medium counting performance region and the poor counting performance, as shown in Figure 2b. The original TSC spectra of these regions are given in Figure 7a, which shows that the current intensity varies considerably in the different regions. The peaks overlap with each other and are difficult to distinguish, so the simultaneous multiple peak analysis (SIMPA) method proposed by Pavlovic [28] was utilized to investigate the trap signatures, namely the activation energy, *E_a_*, the capture cross-section, σ, and the trap density, *N_T_*. The TSC current, *I_TSC_*, of each peak can be estimated as

(5) $$\begin{array}{c} I_{TSC}=N_{T}\mu \tau eAE\mu D_{t}T^{2}\times \exp\left\{-\frac{E_{a}}{kT}-\frac{kD_{t}}{\beta E_{a}}T^{4}\times \exp\left(-\frac{E_{a}}{kT}\right)\times \left[1-4\frac{kT}{E_{a}}+20{\left(\frac{E_{a}}{kT}\right)}^{2}\right]\right\} \end{array}$$

where *μτ* is the mobility-lifetime product of the charge carriers, *e* is the electron charge, *A* is the electrode area, and *E* is the strength of the electric field. *k* is the Boltzmann constant, and β is the heating rate. The trap-dependent coefficient is defined as *D_t_* = 3 × 10^21^ (m*/m_0_) σ. The effective masses of the electrons and holes are 0.14 m_0_ and 0.37 m_0_, with m_0_ being the electron mass. As shown in Figure 7b–d, fourteen main trap peaks are identified from the TSC spectra. The SIMPA fitting results are listed in Table 1, showing the activation energy, *E_a_*, the capture cross-section, σ, the trap density, *N_T_*, and the possible origins of the traps.

The detailed analysis will focus on the peaks related to the A-center, dislocations, Cd vacancies, Te antisites and Te interstitials. Trap T_1_, with the activation energy of 0.106–0.107 eV, can be assigned to the A-center, referring to [29,30,31]. One can see that region A has a lower A-center concentration than region B and C, which is consistent with the PL results. Trap T_2_ located at 0.154–0.159 eV is usually considered to be related to dislocations or dislocation complexes [32]. We found that the concentration of dislocations in the different regions changes little. Traps T_4_, T_8_ and T_10_, with the activation energy range between 0.23 and 0.452 eV, are attributed to the Cd vacancies in the different ionization states, which are considered as acceptor defects in the indium-doped CdZnTe crystals [29,30,31,33]. The fitting results show that region A exhibits a larger concentration of defects associated with the Cd vacancy. The level labeled T_12_ located at 0.57~0.59 eV is assigned to the Te antisites (${\mathrm{Te}}_{\mathrm{cd}}^{2+}$) [34], which is well known as a deep electron trap. The higher ${\mathrm{Te}}_{\mathrm{cd}}^{2+}$ and lower ${\mathrm{In}}_{\mathrm{cd}}^{+}$ concentrations observed in region A should be responsible for the higher resistivity. Trap T_14_, with an activation energy of 0.638~0.642 eV, corresponds to the deep levels of the Te interstitials (Te_i_) [34], which are expected to be deep hole traps with large capture cross-sections. Under high-flux X-ray irradiation, the free holes are easily trapped in the deep-level hole traps to form the positive space charges near the cathode. The presence of positive space charges can strongly interfere with the internal electric field and reduce the effective electric field strength. Therefore, the charge collection efficiency of the detector decreases, which means that the pulse signal amplitude is reduced. When the signal amplitude is lower than the threshold of the ASIC readout system, the detector counting performance deteriorates. Compared to the other regions, region C shows a larger Te_i_ concentration (3.00 × 10^14^ cm^−3^, an order of magnitude higher than the others). We, therefore, suggest that ${\mathrm{Te}}_{i}$-related defects are the key traps affecting photon counting performance. In summary, the region with a good photon counting performance shows a lower A-center and Te_i_ concentration, and a larger ${\mathrm{Te}}_{\mathrm{cd}}^{2+}$ and Cd vacancy-related defect concentration. These results are consistent with the PL results and the inference of the native defect reaction theoretical model.

## 4. Conclusions

The 16-pixel CdZnTe X-ray photon counting detectors with non-uniform counting performances were studied experimentally and theoretically. The effect of deep-level defects on the counting performance is obtained by analyzing the I–V curves, IR optical microscope photography, PL and TSC results. The detectors with a good counting performance show a higher resistivity, which is due to their lower shallow donor ${\mathrm{In}}_{\mathrm{cd}}^{+}$ and higher deep electron trap ${\mathrm{Te}}_{\mathrm{cd}}^{2+}$. The detectors with a good photon counting performance should have a lower A-center and ${\mathrm{Te}}_{i}$ concentration and a larger ${\mathrm{Te}}_{\mathrm{cd}}^{2+}$ and Cd vacancy-related defect concentration. The underlying relationships of resistivity, deep-level defects and the photon counting performance can be explained by the proposed theoretical model of the native defect reaction in indium-doped Cd_0.9_Zn_0.1_Te crystals. The lower ${\mathrm{In}}_{\mathrm{cd}}^{+}$ means that more $V_{\mathrm{cd}}^{2-}$ can remain to react with Te_i_ to form ${\mathrm{Te}}_{\mathrm{cd}}^{2+}$, resulting in an increase in the ${\mathrm{Te}}_{\mathrm{cd}}^{2+}$ concentration and a decrease in the Te_i_ concentration. The deep hole trap Te_i_, with the activation energy of 0.638–0.642 eV, can easily trap holes to form space charges near the cathode which may result in detector polarization. SIMPA fitting results show that the Te_i_ concentration of a poor counting performance region is an order of magnitude higher than the others, and thus we consider Te_i_ to be the key deep-level trap affecting photon counting performance.

## Figures and Tables

**Table 1:** Parameters of the TSC peaks calculated by the SIMPA method. The “↑”, “→” and “↑” represent the increase, invariant and decrease of the trap level compared to the region A, respectively.

Trap	*E_a_*/eV	Region A		Region B		Region C		Origins
		*σ*/cm^2^	*N_T_* /cm^−3^	*σ*/cm^2^	*N_T_* /cm^−3^	*σ*/cm^2^	*N_T_* /cm^−3^	Possible Defect Type
T_1_	0.106~0.107	7.00 × 10^−21^	8.20 × 10^13^	2.00 × 10^−21^	3.22 × 10^14^ ↑	2.00 × 10^−21^	6.40 × 10^14^↑	A center [29,30,31]
T_2_	0.154~0.159	3.75 × 10^−19^	1.23 × 10^15^	2.50 × 10^−20^	3.28 × 10^15^→	6.40 × 10^−20^	1.38 × 10^15^→	Dislocation [32] related
T_3_	0.174~0.179	6.40 × 10^−20^	3.03 × 10^15^	1.20 × 10^−20^	8.00 × 10^15^→	2.80 × 10^−20^	3.50 × 10^15^→	O_Te_^−^V_cd_^−/2−^ [34]
T_4_	0.23~0.235	1.02 × 10^−18^	6.20 × 10^15^	4.10 × 10^−19^	1.10 × 10^15^↓	1.02 × 10^−18^	8.50 × 10^14^↓	V_cd_^−/2−^ [35]
T_5_	0.261~0.265	2.14 × 10^−18^	5.84 × 10^15^	3.14 × 10^−18^	1.00 × 10^14^↓	5.14 × 10^−18^	2.50 × 10^14^↓	Zn related [36]
T_6_	0.294~0.299	3.90 × 10^−18^	3.19 × 10^15^	5.90 × 10^−18^	9.50 × 10^13^↓	5.90 × 10^−18^	7.00 × 10^13^↓	Neutron radiation [37] related
T_7_	0.32~0.325	1.89 × 10^−18^	3.20 × 10^14^	2.89 × 10^−18^	1.55 × 10^14^↓	2.89 × 10^−18^	8.00 × 10^13^↓	Te_Cd_ complex [33]
T_8_	0.39~0.392	1.43 × 10^−17^	2.85 × 10^14^	2.60 × 10^−17^	1.80 × 10^14^↓	1.93 × 10^−17^	9.00 × 10^13^↓	V_cd_ [29]
T_9_	0.398~0.402	1.79 × 10^−18^	4.70 × 10^14^	3.95 × 10^−18^	2.50 × 10^14^→	2.79 × 10^−18^	2.00 × 10^14^→	Te_cd_^0/+^ [29]
T_10_	0.45~0.452	2.00 × 10^−18^	6.90 × 10^14^	6.92 × 10^−18^	2.00 × 10^14^↓	4.00 × 10^−18^	8.50 × 10^13^↓	V_cd_^2−^ [31]
T_11_	0.5~0.52	6.50 × 10^−18^	3.00 × 10^14^	1.50 × 10^−17^	7.80 × 10^13^↓	8.50 × 10^−18^	5.80 × 10^13^↓	Cl related [29]
T_12_	0.57~0.59	4.40 × 10^−17^	6.00 × 10^13^	5.40 × 10^−17^	4.50 × 10^13^↓	4.90 × 10^−17^	8.50 × 10^12^↓	Te_cd_^++^ [34]
T_13_	0.618~0.622	3.16 × 10^−17^	4.20 × 10^13^	1.80 × 10^−17^	4.70 × 10^13^→	3.16 × 10^−17^	3.20 × 10^13^→	Cd_i_^++^ [33]
T_14_	0.638~0.642	2.50 × 10^−17^	3.20 × 10^13^	6.00 × 10^−18^	7.00 × 10^13^↑	8.50 × 10^−18^	3.00 × 10^14^↑	Te_i_ [34]
